# The clinicopathological parameters and prognostic significance of HER2 expression in gastric cancer patients: a meta-analysis of literature

**DOI:** 10.1186/s12957-017-1132-5

**Published:** 2017-03-21

**Authors:** Yu-ying Lei, Jin-yu Huang, Qiong-rui Zhao, Nan Jiang, Hui-mian Xu, Zhen-ning Wang, Hai-qing Li, Shi-bo Zhang, Zhe Sun

**Affiliations:** 1 Department of Surgical Oncology, First Affiliated Hospital of China Medical University, 110001 Shenyang, Liaoning China; 2 Department of Evidence Based Medicine, First Affiliated Hospital of China Medical University, 110001 Shenyang, Liaoning China; 3 International Education School, China Medical University, 110001 Shenyang, Liaoning China

**Keywords:** HER2/neu, Gastric cancer, Prognosis, Meta

## Abstract

**Background:**

Human epidermal growth factor receptor-2 (HER2) is regarded as an important and promising target in the treatment of HER2-positive breast cancers. However, the correlation of clinicopathological characteristics and prognostic significance of HER2 overexpression in gastric cancer patients remains unclear. Our aim was to clarify this issue.

**Methods:**

Embase, PubMed, and the Cochrane Library were searched for relevant articles published up to May 2016. Outcomes of interest contained sex, age, tumor size, tumor site, tumor node metastasis (TNM) stage, distant metastasis, lymph node metastasis, Lauren’s classification, differentiation grade, lymphovascular invasion, neural invasion, and multivariate analysis data for overall survival.

**Results:**

A total of 41 studies of 17,494 gastric cancer patients were identified with HER2 test. HER2 positive rate was 19.07% (95% CI = 9.16, 28.98). There existed statistical significance between HER2 overexpression and patients’ prognosis (RR = 1.47, 95% CI = 1.09, 1.98). Male patients (OR = 1.48, 95% CI = 1.34, 1.65), proximal tumors (OR = 1.25, 95% CI = 1.07, 1.47), intestinal-type tumors (OR = 3.37, 95% CI = 2.54, 4.47), advanced stage cancers (OR = 1.35, 95% CI = 1.10, 1.66), lymph node metastasis (OR = 1.26, 95% CI = 1.14, 1.41), well-differentiated cancers (OR = 1.79, 95% CI = 1.15, 2.76), and distant metastasis (OR = 1.91, 95% CI = 1.08, 3.38) were correlated with higher HER2 expression rates. However, no statistical differences existed in age, tumor size, lymphovascular invasion, or neural invasion. Subgroup analysis revealed that HER2 expression rates reported in articles from Asian (19.52%) countries were quantitatively higher than those from European (16.91%) areas. Results were consistent with those reports that define HER2 status according to trastuzumab for gastric cancer (ToGA) criteria.

**Conclusion:**

This study showed that HER2 overexpression was associated with poor prognosis in gastric cancer patients. HER2 positive rates may be associated with sex, tumor site, TNM staging system, distant metastasis, lymph node metastasis, Lauren’s classification, and differentiation grade in gastric cancer patients. The HER2 expression rate in Asians may be higher than that in Europeans. This study offers a convenient way for doctors to select patients for relevant HER2 detection and following treatment.

**Electronic supplementary material:**

The online version of this article (doi:10.1186/s12957-017-1132-5) contains supplementary material, which is available to authorized users.

## Background

The human epidermal growth factor receptor-2 (HER2) protein is a 185-kDa transmembrane tyrosine kinase (TK) receptor and a member of the epidermal growth factor receptor (EGFR) family [1, 2]. HER2 expression on tumor cells can influence tumor cell proliferation, migration, differentiation, apoptosis, and adhesion [3]. HER2 expression has been found in gastric cancer. Inhibition of the HER2 receptor is a promising treatment target [4, 5] in breast cancers. Trastuzumab is a monoclonal antibody which specifically targets the HER2 protein by directly binding to the extracellular domain of the receptor. A phase 3, open-label randomized controlled trial showed that trastuzumab in combination with chemotherapy exhibits both efficacy and safety for the first-line treatment of advanced gastric cancer with expression of HER2 [6]. In addition, in several clinical trials, trastuzumab in combination with chemotherapy reveals promising antitumor activity with manageable toxic effects [7–10]. Although the outcomes are encouraging, HER2 positive rate in gastric cancer patients is quite low, which means that only a small subgroup of patients can benefit from anti-HER2 target therapy. In consideration of the low positive expression rate of HER2 and the expense of the HER2 examination and anti-HER2 target therapy, therefore, selecting the subgroup of patients with positive HER2 expression in gastric cancer patients is of vital significance.

Recently, a number of studies reported the clinicopathological parameters and prognosis of HER2-positive gastric cancers. Yet, the outcomes of these studies were not uniform [11–15]. Therefore, we conducted a meta-analysis to assess the association between the clinicopathological parameters and prognostic significance of gastric cancer and HER2 expression by performing a pooled analysis of the available literatures. Our aims were to clarify the prognostic significance of HER2 expression and select those clinical parameters specific to patients with gastric cancer expressing high levels of HER2 to help physicians to select patients to undergo more thorough HER2 detection.

## Methods and materials

### Search methods

Search of Embase, the Cochrane Library, and PubMed for relevant articles published up to May 2016, with the next search strategy: “human epidermal growth factor 2” or “HER2” or “erbB-2” or “HER2/neu” combined with “gastric cancer” or “gastric tumor” or “gastric carcinoma.” References of retrieved reviews were manually screened to broaden the search range.

### Inclusion and exclusion criteria with quality analysis

Eligible studies had to fulfill specific criteria to be entered into this analysis. The inclusion criteria were (1) gastric cancer patients; (2) the expression of HER2 was tested by immunohistochemistry (IHC), chromogenic in situ hybridization (CISH), or fluorescence in situ hybridization (FISH); and (3) information on binary clinicopathological factors according to HER2 status or multivariate survival analysis data was provided. Exclusion criteria were (1) conference abstracts, case reports, letters, and reviews without primary data; (2) studies from which relevant information could not be collected; and (3) duplicated publications.

### Assessment of study quality

To evaluate the quality of the final incorporated trials, two investigators completed quality assessment independently with the Newcastle–Ottawa quality assessment scale (NOS) [16]. According to the NOS, studies were accessed on three broad aspects: (1) selection of the study, (2) comparability of the cohort, and (3) confirmation of the outcomes. Each item also has subitems. The full score was nine stars, and any study that obtained five or more stars was considered high quality [16].

### Data extraction

Two independent investigators extracted the information and, through discussion, decided on the basic characteristics and variables to include. The following data were collected: first author’s name, country, publication year, primary antibody, number of participants, HER2 expression rate, and clinicopathological parameters stratified by HER2 status and multivariate analysis data for overall survival.

### Statistical analysis

Stata version 12.0 (Stata Corp, TX) was used to analyze the data. Dichotomous variables were analyzed with odds ratios (OR) and 95% confidence interval (CI). Risk ratios (RR) and 95% CIs of between HER2+ and HER2− were analyzed with random effects model. According to heterogeneity, a random effects model or fixed effects model was selected. *I* ^2^ statistics was used to evaluate heterogeneity [17]. *I* ^2^ < 25% was considered low, and the data was combined using a fixed effects model (the Mantel–Haenszel method), while for *I* ^2^ > 25%, a random effects model (the DerSimonian and Laird method) was performed for data combination. Sensitivity analysis was conducted by removing individual trials from the list and analyzing the degree of change on the overall results to find out sources of heterogeneity. Publication bias was evaluated by funnel plots. A *P* < 0.05 was regarded to be significant statistical difference.

## Results

### Description of trials

**Fig. 1:**
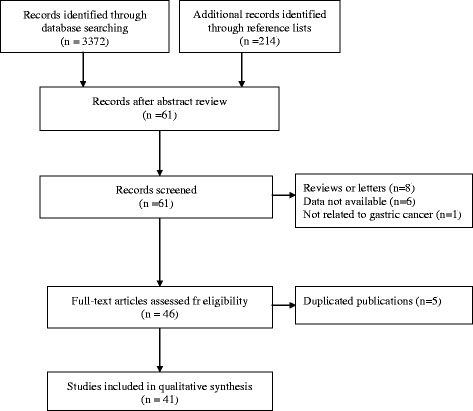
Procedure of study selection

A flow chart describing the process of study selection based on the PRISMA statement (Checklist S1) was shown in Fig. 1 the QUOROM diagram. A final total of 41 articles [11–15, 18–53] of 17,494 participants were included in our study. The basic characteristics of the contained researches were reported in Additional file 1. The details of NOS were described in Additional file 2. Thirty-four Asian countries in our study were included, and the HER2 positive rate was 19.52, (9.32, 29.72). Seven studies of European countries reported the positive rate of HER2 was 16.91, (8.19, 25.63).

### Association of HER2 expression and clinicopathological parameters

The results of our study indicated that HER2 overexpression was associated with sex, tumor site, Lauren’s classification, tumor node metastasis (TNM) stage, lymph node metastasis, differentiation grade, and distant metastasis. However, HER2 overexpression was not related to age, tumor size, lymphovascular invasion, or neural invasion. The detailed information was shown in Table 1.

**Table 1:** Association of HER2 expression and clinicopathological parameters

Clinicopathological parameters	Number of studies	Number of patients	OR (95% CI)	*P*	Heterogeneity	
					*I* ^2^ (%)	*P* value
Age (old vs young)	27	12,800	0.90(0.74,1.10)	0.31	62.9	0.00
Sex (male vs female)	33	15,304	1.48(1.34,1.65)	0.00	21.1	0.14
Tumor size (large vs small)	11	1614	0.83(0.64,1.07)	0.21	13.4	0.32
Tumor site (proximal vs distal)	24	12,853	1.25(1.07,1.47)	0.01	27.4	0.11
Lauren’s classification (intestinal vs diffuse)	30	13,972	3.37(2.54,4.47)	0.00	74.2	0.00
TNM stage (III+IV vs I+II)	24	12,542	1.35(1.10,1.66)	0.01	62.4	0.00
Lymph node metastasis(N+ vs N−)	27	14,075	1.26(1.14,1.41)	0.03	0.0	0.66
Differentiation grade (well vs poor)	28	9086	1.79(1.15,2.76)	0.01	86.6	0.00
Distant metastasis (M+ vs M−)	9	4427	1.91(1.08,3.38)	0.00	54.4	0.03
Lymphovascular invasion (yes vs no)	10	3957	1.30(0.86,1.95)	0.22	62.5	0.00
Neural invasion (yes vs no)	4	1361	0.58(0.24,1.37)	0.21	80.1	0.00

*OR* odds ratio, *CI* confidence interval, *N* node, *M* metastasis

### Risk ratios on overall survival

**Fig. 2:**
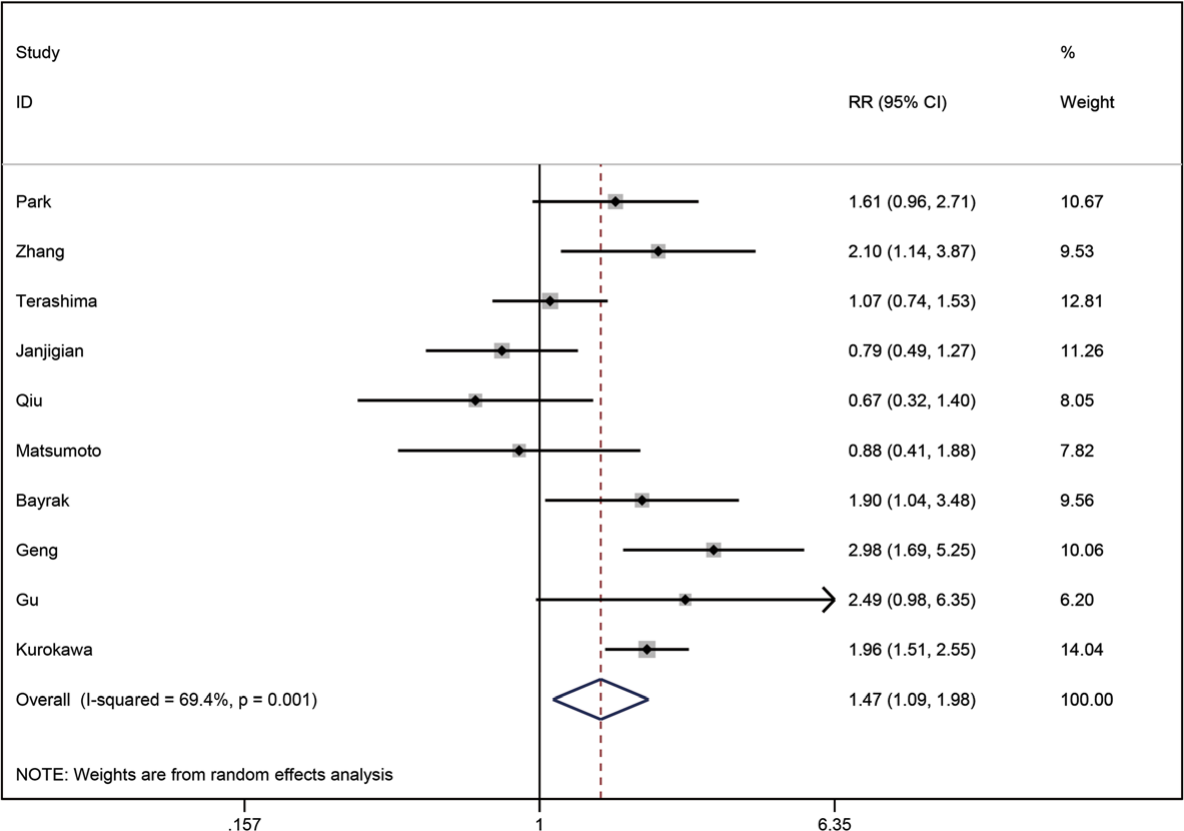
Risk ratios (RRs) and 95% confidence intervals (CIs) in studies assessing the relationship between HER2 expression and overall survival (OS)

Ten of 41 studies reported multivariate survival analysis information on overall survival (OS). Owing to significant heterogeneity, a random effects model was used to combine the data as demonstrated in Fig. 2. The results showed that HER2 overexpression was associated with a poor OS (pooled RR 1.47, 95% CI 1.09–1.98). Significant heterogeneity existed between studies (*I* ^2^ = 69.4%, *P* = 0.001).

### Sensitivity analysis

Trials were removed individually from the list, and the OR value or degree of significance did not substantially change in regard to the risk ratios on overall survival, age, sex, tumor size, tumor site, TNM stage, lymph node metastasis, distant metastasis, differentiation grade, Lauren’s classification, lymphovascular invasion, and neural invasion.

### Publication bias

**Fig. 3:**
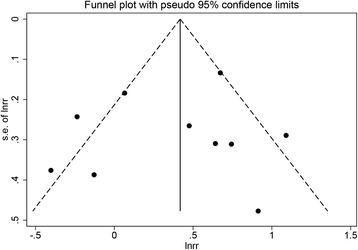
Funnel plot for ten studies included in multivariate analysis of overall survival

To visually assess the publication bias of the researches, funnel plots were performed. The shape of the funnel plots showed symmetry and indicated no significant publication bias (as shown in Fig. 3).

#### Subgroup analysis

By stratifying the trials according to HER2 status by Hoffman validation criteria [54], a further subgroup analysis was conducted. The definition of HER2 positive by Hoffman validation criteria was that tumor samples were scored as 3+ on immunohistochemistry or if they were FISH positive (HER2:CEP17 ratio ≥2). The results obtained from the above analyses are similar to that of the overall analysis (as shown in Table 2).

**Table 2:** Subgroup analysis by defining HER2 status by Hoffman validation criteria

Not fit Hoffman criteria						Fit Hoffman criteria				
Clinicopathological parameters	Number of studies	OR/RR(95% CI)	*P*	Heterogeneity		Number of studies	OR/RR(95%CI)	*P*	Heterogeneity	
				*I* ^2^ (%)	*P* value				*I* ^2^ (%)	*P* value
Sex	18	1.34(1.15, 1.56)	<0.001	0.0	0.716	15	1.63(1.41, 1.89)	<0.001	42.4	0.042
Age	13	1.01(0.72, 1.41)	0.976	72.0	0.000	14	0.78(0.62, 0.99)	0.038	45.6	0.032
Tumor size	7	0.94(0.70, 1.26)	0.692	0.0	0.486	4	0.55(0.32, 0.96)	0.070	12.6	0.330
Tumor site	10	1.06(0.83, 1.37)	0.632	25.3	0.211	14	1.40(1.14, 1.71)	0.001	25.2	0.182
Lauren’s classification	17	3.67(2.47, 5.47)	<0.001	76.2	<0.001	13	3.03(1.95,4.70)	<0.001	73.4	<0.001
Differentiation grade	16	1.24(0.76, 2.05)	0.390	74.8	<0.001	12	2.71(1.66, 4.44)	<0.001	76.8	<0.001
TNM stage (III+IV vs I+II)	14	1.53(1.05,2.23)	0.026	77.4	<0.001	10	1.23(1.04,1.46)	0.014	0.0	0.939
Lymph node metastasis	13	1.15(1.00, 1.33)	0.057	0.0	0.500	14	1.40(1.19, 1.64)	<0.001	0.0	0.847
Distant metastasis	5	2.58(1.00, 6.67)	0.050	64.8	0.023	4	1.52(1.18, 1.95)	0.001	0.0	0.617
Lymphovascular invasion	6	1.18(0.75, 1.85)	0.468	74.1	0.002	4	2.16(0.87, 5.35)	0.096	0.0	0.421
Overall survival	4	1.95(1.38, 2.51)	<0.001	0.0	0.583	6	1.11(0.68, 1.53)	<0.001	70.8	0.004

*OR* odds ratio, *CI* confidence interval, *TNM* tumor, node, metastasis

## Discussion

A meta-analysis was conducted to clarify the correlation between clinicopathological characteristics and prognostic significance of HER2 expression in gastric cancer patients. In current analysis, we included all available data from 41 studies. This study showed that HER2 overexpression was associated with poor prognosis in gastric cancer patients. We also found that gastric cancers in male patients, at proximal sites, of intestinal type, in advanced stages, with lymph node metastasis, that are well differentiated and that have distant metastasis, were more likely to exhibit higher HER2 expression rates. Through subgroup analysis, we also found that HER2 expression rates reported in articles from Asian countries were quantitatively higher than those from European areas. The outcomes of incorporating articles defining HER2 status according to Hoffman validation criteria [6] are similar to that of the overall analysis which indicates that our results are reliable.

HER2, a member of the epidermal growth factor receptor (EGFR) family, is correlated with tumor cell proliferation, migration, differentiation, apoptosis, and adhesion [1–3]. There is growing evidence that HER2 plays an important role in tumorigenesis in gastric cancer [55–57]. Our pooled analysis suggested that HER2 overexpression had played an unfavorable role in the prognosis of gastric cancer. In addition, HER2 positive rates were correlated with TNM stage, distant metastasis, and lymph node metastasis which are consistent with previous researches. Potential clinical benefits of HER2 target therapy may be achieved in the adjuvant treatment for gastric cancer patients with lymph node metastasis and distant metastasis.

Advanced tumor biological behavior, such as distant metastasis and lymph node metastasis, generally indicates poor prognosis. Yet, the results of our pooled analysis indicated that HER2 expression is also strongly related to intestinal-type tumors and well-differentiated gastric cancers, which usually have a better prognosis than diffuse-type tumors or poorly differentiated cancers. These seemingly conflicting observations lead to debate regarding the prognostic significance of HER2 expression. The results of our pooled analysis are in agreement with the outcome of the trastuzumab for gastric cancer (ToGA) trial. The most likely explanation may be that the expression of the HER2 protein in intestinal-type gastric cancers accounts for a small part, and this could not be the only factor included that impacts prognosis [21]. Further researches are needed to interpret this phenomenon.Our study found evidently higher HER2 expression rate in proximal gastric cancers than in distal ones, which was in agreement with the report of the ToGA research [6]. Eric VC et al. suggested that intestinal-type gastric cancers generally occurred more frequently in proximal sites and that different etiologies may play a role in carcinogenesis of cancers from these two sites [58]. This could partly explain different HER2 expression rates depending on tumor location.

Until now, studies exploring HER2 expression in gastric cancers generated conflicting results regarding HER2 expression and its association with clinicopathological parameters. Many factors could result in these inconsistencies, but the most significant ones are most likely the use of different IHC staining methods and the application of inconsistent scoring criteria. Therefore, we performed a subgroup analysis of the researches based on HER2 status according to Hoffman validation criteria, which is the standardized scoring system used for HER2 expression [54]. The results drawn from the subgroup analysis were similar to that of the overall analysis which indicates that our results are reliable.

The low percentage of HER2 expression in gastric cancer patients discourages doctors from detecting HER2 status before starting chemotherapy. In consideration of the expense and side effects of anti-HER2 target therapy, further exploration is necessary to select for those patients who are most likely have high levels of HER2 expression. The clarification of the association between HER2 expression and gastric cancer clinicopathological features offers a convenient way to select for patients that are most likely to have a high level of HER2 expression. This provides principles by which to stratify patients in clinical practice. Our results indicate that patients with advanced stage cancers, distant metastasis, and lymph node metastasis tend to have higher levels of HER2 expression. In addition, these clinicopathological features were associated with aggressive stage cancers and poor prognosis; therefore, our results rationalize the application of HER2-targeting therapy to patients with advanced stage disease that lack effective alternatives.

Several limitations existed in our report. First, the criteria of HER2 status determined by IHC and the primary antibodies varied in studies. However, the results of the studies defining HER2 status by Hoffman validation criteria were similar to that of the overall analysis which indicates that our results are reliable. Second, our results indicated that HER2 overexpression was associated with a poor prognosis. To our knowledge, many factors could affect prognosis. Therefore, we incorporated only the outcomes with the form of multivariate survival analysis to eliminate the potential influence of confounders. Third, biopsy specimen could not represent the whole tumor tissue. Four studies included both resection specimen and biopsy specimen, and three researches did not state in our meta-analysis (Additional file 1). Because none of the included studies stated the details of HER2 expression both in resection specimen or biopsy specimen, we thus could not conduct a subgroup analysis on this issue in the present meta-analyses. Further studies should be conducted to clarify this issue. Fourth, our study was confined to articles written in English, which language bias could not be ruled out.

## Conclusions

In conclusion, our study clarified that HER2 overexpression was associated with poor prognosis in gastric cancer patients. In addition, HER2 overexpression was correlated with the following clinicopathological parameters in gastric cancer patients: sex, tumor site, TNM stage, lymph node metastasis, distant metastasis, differentiation grade, and Lauren’s classification. The HER2 expression rate in Asians may be higher than that in Europeans. This study offers a convenient way for doctors to select patients for relevant HER2 detection and following treatment.

## Additional files

Additional file 1: Baseline characteristics of included studies. (DOCX 29 kb)
Additional file 2: Newcastle–Ottawa Scale (NOS) Table: methodological quality of cohort studies included in the meta-analysis*. (DOC 95 kb)

## Abbreviations

EGFR: Epidermal growth factor
HER2: Human epidermal growth factor receptor-2
IHC: Immunohistochemistry
NOS: Newcastle–Ottawa quality assessment scale
OR: Odds ratio
RR: Risk ratio
TNM: Tumor-node-metastasis
