# Supplementary material for: The clinicopathological parameters and prognostic significance of HER2 expression in gastric cancer patients: a meta-analysis of literature
Source: World J Surg Oncol. 2017 Mar 21;15:68. doi: 10.1186/s12957-017-1132-5 (PMC5359900; doi:10.1186/s12957-017-1132-5)
Supplement: Additional file 1: — Baseline characteristics of included studies. (DOCX 29 kb) [file 12957_2017_1132_MOESM1_ESM.docx]

**Additional file 1 Baseline Characteristics of Included Studies**

| Study | Year | Country | Detection  method | Company | Source | Type | Type  Catalog | Hofferman criteria | NOS | Her2 positive/total | Her2 positive(%) | Resection or bioposy specimen |
| --- | --- | --- | --- | --- | --- | --- | --- | --- | --- | --- | --- | --- |
| Dursun [18] | 1999 | Turkey | NA | NA | NA | MAB | CB 11 | N | 5 | 11/62 | 17.74 | ① |
| Song [19] | 2004 | Korea | NA | Novocastra, UK | NA | MAB | NCL-L-CB11 | N | 5 | 193/738 | 26.2 | ① |
| Tanner [20] | 2005 | Finland | CISH | South San Francisco, CA | NA | NA | NA | N | 7 | 16/131 | 12.2 | ① |
| Park [21] | 2006 | Korea | IHC+FISH | Zymed Labs,  South San Francisco,  CA, USA | Rabbit | PAB | NA | N | 5 | 7/182 | 3.85 | ① |
| Ismail [22] | 2007 | Egypt | IHC | DAKO | Rabbit | MAB | K5204 | N | 5 | 24/93 | 25.8 | ① |
| Kim a [23] | 2007 | Korea | IHC | DAKO, Carpinteria, CA | Rabbit | MAB | A0485 | N | 7 | 56/248 | 22.58 | ① |
| Zhang [24] | 2009 | China | IHC | Dako, Glostrup, Denmark | Rabbit | MAB | NA | N | 6 | 19/102 | 18.63 | ① |
| Ansari [26] | 2011 | Iran | IHC | DAKO USA | NA | MAB | A0485 | N | 5 | 12/100 | 12 | ① |
| Cidon [25] | 2011 | Spain | IHC+FISH | DAKO | Rabbit | MAB | K5207 | F | 5 | 9/120 | 7.5 | ① |
| Kim b [27] | 2011 | Korea | IHC | DAKO, Glostrup, Denmark | Rabbit | MAB | A0485 | N | 5 | 174/1414 | 12.31 | ① |
| Kim c [27] | 2011 | Korea | IHC | DAKO, Glostrup, Denmark | Rabbit | MAB | A0485 | N | 5 | 101/598 | 16.89 | ① |
| Yan [11] | 2011 | China | IHC | NA | NA | NA | NA | N | 7 | 22/145 | 15.17 | ① |
| Kunz [33] | 2012 | America | IHC+FISH | Clin Transl Oncol | Rabbit | MAB | A0485 | F | 7 | 12/99 | 12 | ① |
| Terashima [53] | 2012 | Japan | IHC+FISH | Ventana Medical Systems | NA | NA | NA | F | 5 | 113/829 | 13.6 | ① |
| Janjigian [32] | 2012 | America | IHC+FISH | Ventana PATHWAY | Rabbit | MAB | 4B5 | F | 5 | 78/381 | 20.47 | ③ |
| Chan [28] | 2012 | UK | IHC | PATHWAY | Rabbit | MAB | 4B6 | N | 6 | 20/85 | 24 | ① |
| Sekaran [29] | 2012 | India | IHC+FISH | Dako | Rabbit | PAB | NA | F | 5 | 23/52 | 44.23 | ① |
| Cho [30] | 2012 | Korea | IHC+SISH | PATHWAY Ventana MedicalSystems, Inc.  Tucson, AZ | Rabbit | MAB | 4B5 | F | 5 | 205/2798 | 7.33 | ①+② |
| Dang [31] | 2012 | China | IHC | Dako, Glostrup, Denmark | Rabbit | MAB | A0485 | N | 6 | 37/84 | 44.48 | ① |
| Zhou [34] | 2012 | China | IHC+ISH | PATHWAY | Rabbit | MAB | 4B5 | F | 7 | 27/227 | 11.89 | ① |
| Shan [40] | 2013 | China | IHC | Ventana CONFIRM™ | Rabbit | MAB | 4B5 | N | 5 | 143/1252 | 11.42 | ① |
| Aoyama [35] | 2013 | Japan | IHC+FISH | Dako, Denmark | Rabbit | MAB | A0485 | F | 7 | 10/100 | 10 | ① |
| Bayrak [36] | 2013 | Turkey | IHC | Dako Carpinteria,  CA, USA | Rabbit | MAB | A0485 | N | 5 | 19/46 | 41.3 | ③ |
| Fan [37] | 2013 | China | IHC | Dako, Denmark | Rabbit | MAB | A0485 | N | 5 | 208/957 | 21.73 | ① |
| Gasljevic [38] | 2013 | Croatia | IHC | Dako, Denmark | Rabbit | MAB | A0485 | N | 6 | 77/302 | 25.5 | ① |
| He a [39] | 2013 | China | IHC+FISH | Dako, Denmark | Rabbit | MAB | A0485 | F | 7 | 36/197 | 18.27 | ① |
| Qiu [42] | 2014 | China | IHC+FISH | Ventana Medical Systems,Inc.  Tucson,AZ, USA | NA | NA | 4B5 | F | 5 | 94/838 | 11.22 | ① |
| Aizawa [12] | 2014 | Japan | IHC+ISH | Roche Diagnostics | Rabbit | MAB | 4B5 | F | 5 | 118/1006 | 11.73 | ① |
| Geng [41] | 2014 | China | IHC | DAKO, Carpinteria,CA, USA | Rabbit | MAB | A0485 | N | 7 | 21/110 | 19.09 | ① |
| Son [43] | 2014 | Korea | IHC+FISH | Dako, Glostrup, Denmark | Rabbit | MAB | A0485 | F | 5 | 21/139 | 15.1 | ① |
| Yan [11] | 2014 | China | IHC+FISH | Ventana Medical Systems,Inc.  Tucson, Arizona | Rabbit | MAB | 4B5 | F | 5 | 11/67 | 16.42 | ① |
| He b [46] | 2015 | China | IHC | Gene Technology Company,  Shanghai, China | Rabbit | PAB | GT21007 | N | 7 | 43/498 | 8.63 | ① |
| Madani [49] | 2015 | Iran | IHC | Denmark | NA | NA | A0485 | N | 6 | 79/206 | 38.35 | ③ |
| Gu [45] | 2015 | China | IHC+FISH | Dako,Denmark | NA | NA | NA | F | 6 | 10/92 | 9.2 | ① |
| Kurokawa [13] | 2015 | Japan | IHC+FISH | Ventana Medical Systems,  Tucson, AZ, USA | Mouse | PAB | 4B5 | F | 7 | 180/1148 | 15.7 | ① |
| Laboissiere [47] | 2015 | Brazil | IHC+FISH | Ventana Medical Systems,  Arizona, USA | Rabbit | MAB | 4B5 | F | 5 | 13/124 | 10.5 | ① |
| LI [48] | 2015 | China | IHC | Cell Signaling Technology,Inc. | Rabbit | NA | NA | N | 5 | 20/94 | 21.28 | ① |
| Matsumoto [50] | 2015 | Japan | IHC+FISH | DAKO, Denmark | NA | NA | NA | F | 7 | 24/89 | 26.97 | ①+② |
| Matsusaka [14] | 2015 | Japan | IHC+FISH | Roche Diagnostics,  Tokyo, Japan | Rabbit | MAB | 4B5 | F | 5 | 302/1427 | 21.16 | ①+② |
| Rajagopal [51] | 2015 | Bengaluru | IHC | Dako | Rabbit | PAB | NA | N | 5 | 19/60 | 31.67 | ①+② |
| Tang [15] | 2015 | China | IHC+FISH | Dako Denmark A/S | Mouse | MAB | K5204 | F | 6 | 21/121 | 17.4 | ① |
| Wu [52] | 2015 | China | IHC | Dako, Glostrup, Denmark | NA | NA | NA | N | 7 | 32/133 | 24.06 | ① |

IHC, immunohistochemistry; FISH, fluorescence in situ hybridization; CISH, chromogenic in situ hybridization; NA, not available; MAB, monoclonal antibody; PAB, polyclonal antibody; NOS, Newcastle-Ottawa scale; N, not fit; F, fit; ①, resection specimen; ②, bioposy specimen; ③, not report
