# Supplementary material for: The clinicopathological parameters and prognostic significance of HER2 expression in gastric cancer patients: a meta-analysis of literature
Source: World J Surg Oncol. 2017 Mar 21;15:68. doi: 10.1186/s12957-017-1132-5 (PMC5359900; doi:10.1186/s12957-017-1132-5)
Supplement: Additional file 2: — Newcastle–Ottawa Scale (NOS) Table: methodological quality of cohort studies included in the meta-analysis*. (DOC 95 kb) [file 12957_2017_1132_MOESM2_ESM.doc]

**Additional file 2.** Newcastle-Ottawa Scale (NOS) Table: Methodological Quality Of Cohort Studies Included In The Meta-Analysis*

| **Study** | **Representativeness**  **of the exposed cohort** | **Selection of the unexposed**  **cohort** | **Ascertainment**  **of exposure** | **Outcome of interest**  **not present**  **at start of study** | **Control for**  **important factor or additional factor** | **Assessment of outcome** | **Follow-up**  **long enough for outcomes**  **to occur** | | **Adequacy of**  **follow-up**  **of cohorts** | **Total quality**  **scores** |
| --- | --- | --- | --- | --- | --- | --- | --- | --- | --- | --- |
| Dursun [18] | - | - | * | * | - | * | * | * | | 5 |
| Song [19] | - | - | * | * | - | * | * | * | | 5 |
| Tanner [20] | - | - | * | * | ** | * | * | * | | 7 |
| Park [21] | - | - | * | * | - | * | * | * | | 5 |
| Ismail [22] | - | - | * | * | - | * | * | * | | 5 |
| Kim a [23] | - | - | * | * | ** | * | * | * | | 7 |
| Zhang [24] | - | - | * | * | * | * | * | * | | 6 |
| Ansari [26] | - | - | * | * | - | * | * | * | | 5 |
| Cidon [25] | - | - | * | * | - | * | * | * | | 5 |
| Kim b [27] | - | - | * | * | - | * | * | * | | 5 |
| Kim c [27] | - | - | * | * | - | * | * | * | | 5 |
| Yan [11] | - | - | * | * | ** | * | * | * | | 7 |
| Kunz [33] | - | - | * | * | ** | * | * | * | | 7 |
| Terashima [53] | - | - | * | * | - | * | * | * | | 5 |
| Janjigian [32] | - | - | * | * | - | * | * | * | | 5 |
| Chan [28] | - | - | * | * | * | * | * | * | | 6 |
| Sekaran [29] | - | - | * | * | - | * | * | * | | 5 |
| Cho [30] | - | - | * | * | - | * | * | * | | 5 |
| Dang [31] | - | - | * | * | * | * | * | * | | 6 |
| Zhou [34] | - | - | * | * | ** | * | * | * | | 7 |
| Shan [40] | - | - | * | * | - | * | * | * | | 5 |
| Aoyama [35] | - | - | * | * | ** | * | * | * | | 7 |
| Bayrak [36] | - | - | * | * | - | * | * | * | | 5 |
| Fan [37] | - | - | * | * | - | * | * | * | | 5 |
| Gasljevic [38] | - | - | * | * | * | * | * | * | | 6 |
| He a [39] | - | - | * | * | ** | * | * | * | | 7 |
| Qiu [42] | - | - | * | * | - | * | * | * | | 5 |
| Aizawa [12] | - | - | * | * | - | * | * | * | | 5 |
| Geng [41] | - | - | * | * | ** | * | * | * | | 7 |
| Son [43] | - | - | * | * | - | * | * | * | | 5 |
| Yan [11] | - | - | * | * | - | * | * | * | | 5 |
| He b [46] | - | - | * | * | ** | * | * | * | | 7 |
| Madani [49] | - | - | * | * | * | * | * | * | | 6 |
| Gu [45] | - | - | * | * | * | * | * | * | | 6 |
| Kurokawa [13] | - | - | * | * | ** | * | * | * | | 7 |
| Laboissiere [47] | - | - | * | * | - | * | * | * | | 5 |
| LI [48] | - | - | * | * | - | * | * | * | | 5 |
| Matsumoto [50] | - | - | * | * | ** | * | * | * | | 7 |
| Matsusaka [14] | - | - | * | * | - | * | * | * | | 5 |
| Rajagopal [51] | - | - | * | * | - | * | * | * | | 5 |
| Tang [15] | - | - | * | * | * | * | * | * | | 6 |
| Wu [52] | - | - | * | * | ** | * | * | * | | 7 |

* A study could be awarded a maximum of one star for each item except for the item Control for important factor or additional factor. The definition/explanation of each column of the Newcastle-Ottawa Scale is available at <http://www.ohri.ca/programs/clinical_epidemiology/oxford.htm>.
